# Of monkeys and men: Impatience in perceptual decision-making

**DOI:** 10.3758/s13423-015-0958-5

**Published:** 2015-10-30

**Authors:** Udo Boehm, Guy E. Hawkins, Scott Brown, Hedderik van Rijn, Eric-Jan Wagenmakers

**Affiliations:** Department of Experimental Psychology, University of Groningen, Grote Kruisstraat 2/1, 9712 TS Groningen, The Netherlands; Amsterdam Brain and Cognition Center, University of Amsterdam, 1018 WS Amsterdam, The Netherlands; School of Psychology, University of Newcastle, Callaghan, NSW 2308 Australia; Department of Psychology, University of Amsterdam, 1018 XA Amsterdam, The Netherlands

**Keywords:** Decision-making, Drift diffusion model, Collapsing bounds, Reward rate maximization, Single-cell recordings

## Abstract

For decades sequential sampling models have successfully accounted for human and monkey decision-making, relying on the standard assumption that decision makers maintain a pre-set decision standard throughout the decision process. Based on the theoretical argument of reward rate maximization, some authors have recently suggested that decision makers become increasingly impatient as time passes and therefore lower their decision standard. Indeed, a number of studies show that computational models with an impatience component provide a good fit to human and monkey decision behavior. However, many of these studies lack quantitative model comparisons and systematic manipulations of rewards. Moreover, the often-cited evidence from single-cell recordings is not unequivocal and complimentary data from human subjects is largely missing. We conclude that, despite some enthusiastic calls for the abandonment of the standard model, the idea of an impatience component has yet to be fully established; we suggest a number of recently developed tools that will help bring the debate to a conclusive settlement.

## Introduction

Most modern accounts of human and monkey decision-making assume that choices involve the gradual accumulation of noisy sensory evidence from the environment in support of alternative courses of action. When the evidence in favor of one response option accrues to a threshold quantity a decision is reached and the corresponding action is initiated (Ratcliff & Smith, 2004). This successful class of models is referred to as sequential sampling models. In the popular random dot motion task (Britten, Shadlen, Newsome, & Movshon, 1992), for example, the decision maker is presented with a cloud of pseudo-randomly moving dots that give the impression of coherent motion to the left or right, and the decision maker must determine the direction of movement. In this example, the models assume that noisy evidence for rightward and leftward motion is integrated over time until the decision threshold for a “right” or “left” response is crossed.

Sequential sampling models are most simply instantiated as random walk models, which assume that evidence and time are measured in discrete steps (Ashby, 1983; Edwards, 1965; Heath, 1981; Stone, 1960). The generalization of the random walk to continuous evidence and time leads to a class of models with more favorable mathematical and empirical properties known as drift diffusion models (DDM; Ratcliff, 1978, Ratcliff & McKoon, 2008). These models make predictions for the response times and accuracy rates for each of the possible actions (Smith, 1995).

For almost 40 years, the DDM has successfully accounted for data from a vast range of perceptual decision-making paradigms. In almost all of these applications, the DDM assumes that decision makers set the height of the decision threshold before a decision trial commences, and that this threshold is constant throughout the decision process. This assumption implies that the decision maker requires the same amount of evidence to trigger a decision regardless of how long the decision takes; the decision criterion does not change over time. With this assumption, the standard DDM has explained not only behavioral output of the decision-making process, namely response time and decision accuracy, but also physiological measures related to gradually accumulating evidence from the environment such as EEG, MEG, and fMRI in humans (Ratcliff, Philiastides, & Sajda, 2009; Philiastides & Sajda, 2006; Mulder, Wagenmakers, Ratcliff, Boekel, & Forstmann, 2012) and single-cell recordings in monkeys (Ratcliff, Hasegawa, Hasegawa, Smith, & Segraves, 2007; Huk & Shadlen, 2005; Purcell et al., 2010, 2012).

Recently, the assumption of a fixed threshold in the standard DDM has been challenged. It has been proposed that decision makers become increasingly impatient as the decision time increases, and therefore steadily decrease the amount of evidence required to trigger a decision. Such a decreasing decision criterion can be implemented in the DDM in two ways: decision thresholds could decrease over time (Bowman, Kording, & Gottfried, 2012; Ditterich, 2006a, b; Drugowitsch, Moreno-Bote, Churchland, Shadlen, & Pouget, 2012; Gluth, Rieskamp, & Büchel, 2012, 2013a; Milosavljevic, Malmaud, & Huth, 2010), or the incoming evidence could be multiplied by an urgency signal that increases in strength over time (Cisek, Puskas, & El-Murr, 2009; Deneve, 2012; Hanks, Mazurek, Kiani, Hopp, & Shadlen, 2011; Thura, Beauregard-Racine, Fradet, & Cisek, 2012, 2014), thus increasingly amplifying moment-to-moment fluctuations in evidence. Both approaches increase the likelihood of the accumulated evidence crossing one of the decision thresholds as time passes. The similarities in the predictions of these two extensions to the DDM outweigh their differences, but both differ markedly to the standard DDM (Hawkins, Wagenmakers, Ratcliff, & Brown, 2015). We will therefore discuss both extensions together and refer to this class of models as those implementing a dynamic decision criterion as compared to the standard DDM, which implements a static decision criterion.

Here, we review the theoretical motivations for dynamic decision criteria and the behavioral and neural evidence in support of these proposals. Dynamic DDMs have received some empirical support (Churchland, Kiani, & Shadlen, 2008; Ditterich, 2006b; Gluth et al., 2012, 2013a; Hanks et al., 2011; Milosavljevic et al., 2010) and have been incorporated as a standard assumption in some neural network models of decision-making (Standage, You, Wang, & Dorris, 2011; Huang & Rao, 2013; Rao, 2010). Nevertheless, empirical and theoretical questions that might have a profound impact on the generality of the dynamic decision criterion have not been adequately addressed. Model-based studies of perceptual decision-making have provided strong support for the existence of a dynamic criterion in a range of experimental tasks, but the evidence is less clear in other situations. Future research must determine how to quantify the amount of support the data lend to models with dynamic compared to static decision criteria in situations where the evidential support is currently ambiguous.

### Collapsing thresholds and urgency gating

**Fig. 1:**
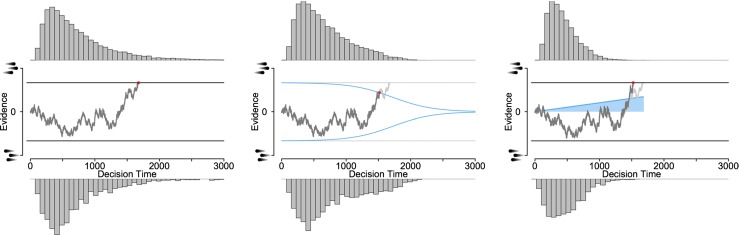
Three versions of the drift diffusion model for a two-alternative forced choice paradigm, such as the random dot motion task. The upper decision threshold corresponds to a “right” decision and the lower threshold corresponds to a “left” decision. The drift rate is positive in this example (the evidence process drifts upward) indicating that the correct response is “the dots are moving to the right”. The left panel shows the standard DDM with static decision thresholds where a choice is made when the accumulated evidence reaches one of the two thresholds. The middle panel shows a DDM with collapsing thresholds that gradually move inward so that less evidence is required to trigger a decision as time passes (blue lines). This decision policy predicts shorter decision times than the DDM with static thresholds when faced with weak evidence (i.e., a low drift rate) as it partially truncates the negatively skewed distribution of response times. The right panel shows a DDM with an urgency gating mechanism. The accumulated evidence is multiplied with an urgency signal that increases with increasing decision times (*blue line*). This decision policy again predicts shorter decision times than the DDM with static thresholds but also increased variability as moment-to-moment variations in the accumulated evidence are also multiplied

Dynamic diffusion models assume that the amount of evidence required to trigger a decision fluctuates over time. Across modeling frameworks such as neural networks and mathematical models, the mechanisms underlying dynamic decision criteria are generally implemented in one of two forms: collapsing thresholds or urgency gating (Fig. 1).

Models with collapsing thresholds assume that decision thresholds move inward as decision duration increases (Bowman et al., 2012; Drugowitsch et al., 2012; Gluth et al., 2013a, b; Milosavljevic et al., 2010). This results in a shortening of the slow decisions in cases where only little information is provided by the environment, thus reducing the right tail of the response time distribution in comparison to the standard DDM with static decision criteria (Ditterich, 2006a).

Models with an urgency gating mechanism assume a static decision threshold but that the incoming evidence is multiplied by an urgency signal that increases in strength over time (Cisek et al., 2009; Deneve, 2012; Huang & Rao, 2013; Niyogi & Wong-Lin, 2013; Rao, 2010; Standage et al., 2011; Thura et al., 2012; Thura & Cisek, 2014). Similar to collapsing thresholds, urgency signals predict faster decisions when the environment only weakly informs the decision. At the same time, the urgency signal increasingly enhances moment-to-moment fluctuations in accumulated evidence as time passes, leading to more variability in the final decision compared to the standard DDM.

One variation of the urgency gating model uses an additive gain mechanism; the evidence is added to, rather than multiplied by, an urgency signal (Hanks et al., 2011, 2014). The predictions of the additive urgency model are very similar to those of the collapsing thresholds model because the additive urgency signal speeds up decisions if only little information is provided by the environment, resulting in a shortened right tail of the response time distribution.

## Why a dynamic component?

In the early history of sequential sampling models, dynamic evidence criteria were introduced to improve model fit to data. For example, models with a dynamic decision criterion were required to account for fast but erroneous responses in discrimination tasks with high time pressure (Swensson & Thomas, 1974), detection tasks with stimuli rapidly presented against noisy backgrounds (Heath, 1992) and, in some cases, trading decreasing decision accuracy for faster responses (Pike, 1968). Although some modern arguments for dynamic decision criteria are grounded in improving model fit to data (Ditterich, 2006b), most are supported by elaborate theoretical considerations.

### Maximizing reward rate

One motivation for dynamic decision criteria is that decision makers strive to maximize the total reward gained or, equivalently, minimize losses, across a sequence of decisions. For instance, in deferred decision-making tasks the observer sequentially purchases discrete units of information that provide evidence in favor of one or another course of action. With a known maximum number of units that can be purchased, and each additional unit bearing a larger cost than the previous unit, expected loss is minimized with a decision criterion that decreases as the number of purchased evidence units increases (Rapoport & Burkheimer, 1971), and humans appear to qualitatively employ this strategy (Pitz, 1968; Busemeyer & Rapoport, 1988; Wallsten, 1968).

Reward has also been a motivating factor in recent dynamic DDMs, often in the form of maximizing reward rate, that is, the expected number of rewards per unit of time (Gold, Shadlen, & Sales, 2002). For instance, when the decision maker is rewarded for a correct choice, under some environmental conditions reward rate is maximized by adopting decision criteria that decrease over time (Thura et al., 2012; Standage et al., 2011). Rather than maximizing reward rate per se, related approaches have considered maximization of the expected total sum of future rewards (Huang & Rao, 2013; Rao, 2010) and trading the reward obtained for a correct decision with the physiological cost associated with the accumulation of evidence (Drugowitsch et al., 2012). Physiological costs are assumed to increase with decision time, leading to a growing urgency to make a decision and hence a decreasing dynamic decision criterion.

Interestingly, most studies proposing that maximizing reward rate gives rise to a dynamic decision criterion do not experimentally manipulate or control rewards and/or punishments. For example, in one study human participants’ remuneration was independent of their performance in a random dot motion task, yet the model the authors aimed to support assumes that humans maximize reward rate by considering the physiological cost of accumulating additional sensory evidence (Drugowitsch et al., 2012). Similarly, another study used an expanded judgment task (Vickers, 1979) where coins stochastically flipped from a central pool to a left or a right target, and the participant was to decide whether the left or the right target accumulated more coins (Cisek et al., 2009). In the experiment by Cisek et al., participants were informed that the experiment would continue until a preset number of correct responses had been achieved; this instruction may have led participants to minimize time on task (and hence maximize reward rate). Although Cisek et al. reported data that were qualitatively consistent with predictions of a dynamic DDM, the lack of an experimental manipulation of reward rates leaves it open whether it was indeed reward rate maximization that caused the decision maker to adopt a dynamic decision criterion.

#### Reward rate maximization in environments with stable signal-to-noise ratio

Empirical support that decision makers can maximize reward rate when the task structure encourages such a strategy primarily comes from fits of DDMs with static decision criteria. These studies demonstrate that participants set their decision criteria in a manner consistent with the threshold settings that maximize reward rate (Balci et al., 2011; Bogacz, Brown, Moehlis, Holmes, & Cohen, 2006; Simen et al., 2009). However, two studies also found evidence that some participants, at least when not fully acquainted with the decision task, favored accuracy over reward rate maximization by setting their criterion higher than the optimal value for reward rate maximization (Bogacz et al., 2006; Balci et al., 2011; Starns & Ratcliff, 2010, 2012). These findings suggest that humans might maximize a combination of reward rate and accuracy rather than reward rate per se (Maddox & Bohil, 1998). Furthermore, the fact that both studies used a static DDM means that it remains unclear how close human decision makers’ static criteria were to the threshold settings that maximize reward rate compared to a model with dynamic criteria. This seems particularly important since the gain in reward rate obtained with a dynamic compared to a static criterion might be small (Ditterich, 2006b).

#### Reward rate maximization in environments with variable signal-to-noise ratio

Whether humans and monkeys do indeed optimize reward rate or implement dynamic decision criteria might depend crucially on the signal-to-noise ratio of the decision environment, often described as the difficulty of the decision (e.g., coherence in the random dot motion task, or word frequency in a lexical decision task). In particular, decision makers might rely on a dynamic criterion when the signal-to-noise ratio is poor. With a weak signal one must accumulate evidence over an extended period to make an accurate decision. To avoid the prohibitively high costs associated with extended accumulation, decision makers could adopt a dynamically decreasing decision threshold (Drugowitsch et al., 2012; Hanks et al., 2011). As decision duration increases, decision makers should be increasingly willing to sacrifice accuracy for a shorter decision time, so they can engage in a new decision with a potentially more favorable signal-to-noise ratio and hence a better chance of obtaining a reward.

When the signal-to-noise ratio varies from one decision to the next, setting a static criterion prior to decision onset is suboptimal because the occurrence of a weak signal would lead to prohibitively long decision times (i.e., the decision criterion is too high) or an unacceptably high error rate (i.e., the signal-to-noise ratio is too low; Shadlen & Kiani, 2013). Relatively few studies have tested this issue empirically. For example, it has been demonstrated that when signal strength varies across trials from pure noise to very strong signals, dynamic DDMs provide a better account of human and monkey behavioral data than models with static decision criteria (Bowman et al., 2012; Drugowitsch et al., 2012; Hanks et al., 2011, 2014). However, a recent meta-analysis suggests that models with dynamic decision criteria do not necessarily provide the best account of behavioral data obtained in environments with variable signal-to-noise ratios across decisions.

#### Behavioral evidence for static and dynamic criteria in drift diffusion models

**Fig. 2:**
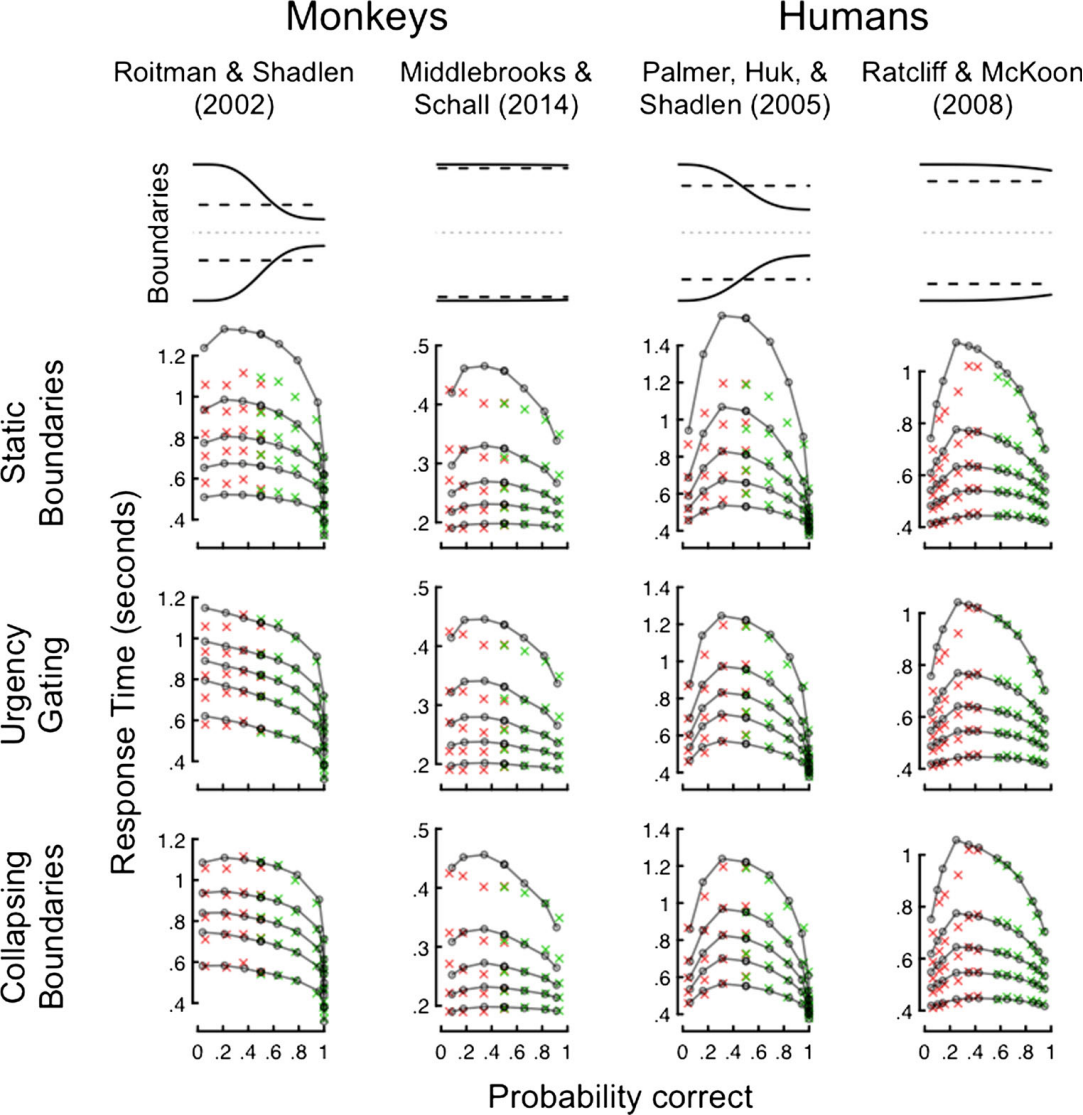
DDMs with static and dynamic decision criteria fitted to four data sets (subset of results reported in Forstmann, et al., 2015). Column names cite the original data source, where example data sets from non-human primates and humans are shown in the left two and right two columns, respectively. The upper row shows the averaged estimated collapsing (*solid lines*) and static (*dashed lines*) thresholds across participants. The second, third and fourth rows display the fit of the static thresholds, urgency gating, and collapsing thresholds models to data, respectively. The *y*-axes represent response time and *x*-axes represent probability of a correct choice. Green and red crosses indicate correct and error responses, respectively, and black lines represent model predictions. Vertical position of the crosses indicate the 10th, 30th, 50th, 70th, and 90th percentiles of the response time distribution. When the estimated collapsing and static thresholds markedly differed (first and third columns), the DDMs with dynamic decision criteria provided a better fit to data than the DDM with static criteria. When the collapsing thresholds were similar to the static thresholds (second and fourth columns), the predictions of the static and dynamic DDMs were highly similar, which indicates the extra complexity of the dynamic DDMs was not warranted in those data sets. For full details see Hawkins, Forstmann, et al., 2015

When quantitative models are proposed they are typically tested against only a few data sets as proof-of-concept evidence for the validity of the model. This approach is a prerequisite for theoretical progress but it necessarily restricts the generality of the model by testing it across only a narrow range of experimental tasks, procedures, and even species. Recently, we quantitatively compared static and dynamic DDMs in a large-scale survey of behavioral data sets that spanned a range of experimental paradigms and species, and across independent research laboratories (Hawkins, Forstmann, Wagenmakers, Ratcliff, & Brown, 2015). Whether quantitative model selection indices indicated that humans or non-human primates used static or dynamic decision criteria depended on specific experimental procedures or manipulations. For instance, decision makers were more likely to adopt dynamic decision criteria after extensive task practice (e.g., left column in Fig. 2) or when the task structure imposed a delayed feedback procedure (delay between stimulus onset and the timing of rewards for correct decisions, middle right column in Fig. 2). Further targeted experimentation combined with rigorous quantitative model comparison is required to clarify when and why decision makers employ static or dynamic response thresholds.

#### Inferring optimal decision criteria from the signal-to-noise ratio

The suggestion that dynamic decision criteria maximize reward rate in environments with a poor signal-to-noise ratio implicitly raises the question of how decision makers infer the current signal strength. If the signal remains constant throughout the decision process, a simple solution is to incorporate elapsed time as a proxy for signal strength into the decision variable (Hanks et al., 2011), because more time will pass without the decision variable crossing one of the two thresholds. There is even some evidence that certain neurons in the lateral intraparietal (LIP) area provide a representation of elapsed time that can be incorporated into the formation of the decision variable (Churchland et al., 2008, 2011; Janssen & Shadlen, 2005; Leon & Shadlen, 2003). It is less clear how the brain handles signals that change in strength throughout the decision process. The decision maker would need to maintain and update an estimate of the instantaneous rate of information conveyed by the information source. A Bayesian estimate might be obtained from changes in the firing rates of neurons representing the evidence in early visual areas (Deneve, 2012). Empirical investigations of how such an estimate of the signal strength is obtained and incorporated into the decision variable are lacking.

How should a time-variant signal-to-noise ratio inform threshold settings? A static decision criterion is highly insensitive to signals that vary throughout a trial, increasing the probability of an erroneous decision. A sensible approach might be to place greater weight on information presented later in the decision process, which can be achieved with a dynamic decision criterion. The distance between a dynamic decision threshold and the decision variable will decrease as more time passes, irrespective of the current state of the evidence accumulation process. This increases the likelihood of momentary sensory evidence leading to a threshold crossing (Cisek et al., 2009; Deneve, 2012; Thura et al., 2012).

In support of this proposal, evidence that varies throughout a trial can induce prominent order effects. For example, when a bias for a response option appears early in a trial it does not influence human and monkey decision times (Cisek et al., 2009; Thura et al., 2012, 2014; although one study found an influence of early evidence Winkel, Keuken, Van Maanen, Wagenmakers, & Forstmann, 2014), but leads to faster and more accurate decisions when it is presented later in the decision process (Sanders & Ter Linden, 1967), meaning that later evidence had a larger influence on the final decision. Notably however, recency effects are not a universal response to a variable signal. Rather, some participants show the opposite reaction, placing increased weight on early information (Usher & McClelland, 2001; Summerfield & Tsetsos, 2012; Resulaj, Kiani, Wolpert, & Shadlen, 2009). The interpretation of studies finding a recency effect is further complicated by the fact that these studies did not compare environments with variable versus static signals. Therefore, it remains unclear whether variation in the signal causes decision makers to adopt a decreasing dynamic criterion.

Taken together, formal analyses indicate that whether static or dynamic decision criteria are the optimal decision strategy depends critically on whether two components of the decision environment are fixed or variable within- and between-trials: the reward for a correct choice and the signal-to-noise ratio. When both the reward for a correct decision and the signal-to-noise ratio are constant across trials, the static thresholds DDM maximizes reward rate (for an extensive review see Bogacz et al., 2006). When the reward for a correct decision is constant over trials and the signal-to-noise ratio varies between trials, a dynamic decision criterion maximizes reward rate (Drugowitsch et al., 2012; Miller & Katz, 2013; Thura et al., 2012, 2014; Ditterich, 2006a). Finally, when the reward varies between or even within trials (as is often the case in economic decision-making), dynamic decision criteria are optimal (Rapoport & Burkheimer, 1971; Frazier & Yu, 2008).

It remains unclear however, whether human and monkey decision makers actually use the optimal threshold settings under the different environmental conditions. Although there is some evidence that humans can optimize reward rate there does not seem to be a consensus yet as to whether reward rate maximization is the only goal. Most studies that suggest reward rate as the cause of a dynamic decision criterion do not actually manipulate or even control rewards. However, a number of studies that systematically manipulated rewards showed that increasing sampling costs can cause a dynamic criterion (Pitz, 1968; Busemeyer & Rapoport, 1988; Wallsten, 1968). Another consideration is that it is complicated to establish a link between a dynamic criterion and reward rates across species. While behavioral studies in humans abound, equivalent data from monkeys is scarce, and the two sets of findings are not necessarily comparable.

### Decision-making in the brain

**Fig. 3:**
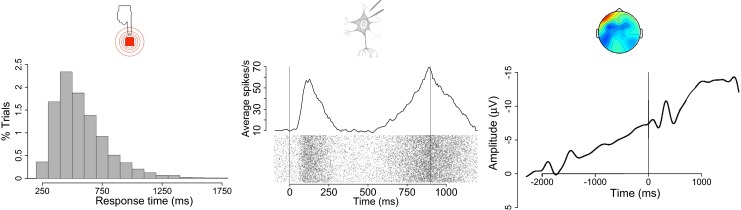
Behavioral and physiological variables used in the evaluation of DDMs. The left panel shows a response time distribution, the classic behavioral variable against which DDMs are tested. The middle panel shows activity patterns of individual neurons (*bottom*) and the average firing rates of such a neuron population (*top*). The right panel shows an averaged EEG waveform, which reflects the aggregate activity of large neuron ensembles in the human cortex. Model comparisons based on behavioral outcomes such as response time distributions are limited in their ability to discriminate between models with different process assumptions but similar behavioral predictions. Physiological measurements such as single-cell recordings in primates and EEG recordings in humans allow for thorough evaluation of the process assumptions underlying candidate models. A question that still remains unanswered is how physiological measurements at different levels of aggregation (i.e., single neurons vs. large neuron populations) relate to each other, and the degree to which they constrain process models (full behavioral and EEG data reported in Boehm, Van Maanen, Forstmann, & Van Rijn, 2014; single-cell data were generated using a Poisson model)

Even though sequential sampling models make elaborate assumptions about the processes underlying decision-making, behavioral studies – the most common source of data for model comparison – cannot take advantage of this wealth of discriminating information. In fact, different models often make indiscernibly similar behavioral predictions and thus only data on the physiological implementation of the decision process (Fig. 3) might allow researchers to discriminate amongst models with dynamic and static decision criteria (Ditterich, 2010; Purcell et al., 2010; Jones & Dzhafarov, 2014).

There is considerable evidence for the neural implementation of DDMs, for instance from single-cell recordings of monkeys performing experimental decision-making tasks (Forstmann, Ratcliff, & Wagenmakers, in press). Neurons in area LIP (Churchland et al., 2008; Gold & Shadlen, 2007; Hanks et al., 2011, 2014; Huk & Shadlen, 2005; Roitman & Shadlen, 2002; Shadlen & Newsome, 2001; N. W. D. Thomas & Paré, 2007) and FEF (Hanes & Schall, 1996; Heitz & Schall, 2010; Purcell et al., 2012, 2012), amongst others (Ratcliff et al., 2011), show patterns of activity that closely resemble the evidence accumulation process proposed in DDMs, and even correlate with the monkeys’ observed decisions. For instance, when non-human primates made decisions in a random dot motion task with a variable signal-to-noise ratio across trials, a DDM with a dynamic compared to static decision criterion provided a better fit to the distribution of response times (Ditterich, 2006b; Hanks et al., 2011, 2014) and the firing patterns of individual neurons (Ditterich, 2006a; Hanks et al., 2014; although other studies show good correspondence between physiologically informed DDMs with a static decision criterion and behavioral data; Purcell et al., 2010, 2012, Heitz & Schall, 2012). Simulation-based studies of neuronal networks have provided convergent evidence: dynamic decision criteria lead to greater stability in biologically plausible networks (Cain & Shea-Brown, 2012; Miller & Katz, 2013; Niyogi & Wong-Lin, 2013) and the stereotypical time course of neural activity in LIP neurons (Niyogi & Wong-Lin, 2013).

Another method of contrasting DDM decision processes with physiological data relies on measurements of the aggregated activity of large neuron ensembles in human subjects, such as EEG, MEG, and fMRI. This line of research is motivated on the assumption that the activity of neuron populations control behavior, not single neurons (Deco, Rolls, & Romo, 2009; Lo, Boucher, Paré, Schall, & Wang, 2009; Smith, 2010; Wang, 2002; Zandbelt, Purcell, Palmeri, Logan, & Schall, 2014). Therefore, such measures of aggregated neuronal activity might provide more insight into the decision criterion underlying human decision-making. However, due to the noisy nature of non-invasive measures such as EEG and fMRI, it is challenging to directly identify physiological correlates of the evidence accumulation process (Kelly & O’Connell, 2013; O’Connell, Dockree, & Kelly, 2012; Wyart, de Gardelle, Scholl, & Summerfield, 2012). An indirect way of obtaining EEG measures of the current state of the decision-making process might be to monitor the accumulated evidence as it is propagated down the processing stream toward motor output structures (Donner, Siegel, Fries, & Engel, 2009; Siegel, Engel, & Donner, 2011; Heekeren, Marrett, & Ungerleider, 2008). The activity of these motor structures can then easily be identified in motor related potentials (Leuthold & Jentzsch, 2002; Lang et al., 1991). For example, human participants making decisions under either high or low sampling costs showed a faster increase in motor-related EEG activity if sampling costs were high, a pattern which was best accounted for by a model with a dynamic decision criterion (Gluth et al., 2013a, b; although other studies reported a good fit between EEG data and a DDM with a static decision criterion Cavanagh et al., 2011, Martin, Huxlin, & Kavcic, 2010, Van Vugt, Simen, Nystrom, Holmes, & Cohen, 2012). A related fMRI study showed similar results (Gluth et al., 2012).

Taken together, physiological evidence from monkeys, and to a lesser extent from humans, supports the suggestion of a dynamic decision criterion. As time passes, less evidence is needed for decision commitment because an urgency signal increasingly drives neural activity toward the decision threshold. However, comparisons of such neural activity patterns and generalizations across species are complicated because measurements differ in a number of ways. Not only is the mapping between primate and human brain activity uncertain (Mantini et al., 2012; Orban, Van Essen, & Vanduffel, 2004; Petrides, Tomaiuolo, Yeterian, & Pandya, 2012) but neural activity is often measured with different temporal and spatial resolution and on vastly different scales. While single-cell recordings in monkeys are obtained with great temporal resolution and spatial resolution, physiological recordings in humans usually represent a tradeoff between either high spatial resolution with low temporal resolution (i.e., fMRI), or high temporal resolution with low spatial resolution (i.e., EEG). Moreover, the activity of individual neurons may or may not impose strong constraints on activity patterns observable at the level of neuron populations. Ensembles of individual neurons that can be adequately described by a DDM with a static decision criterion exhibit combined activity patterns that are best described by a DDM with a static decision criterion, as shown in recent theoretical work (Zandbelt et al., 2014). However, similar theoretical studies outlining the constraints individual accumulators with a dynamic decision criterion impose on the combined activity of neuron populations are lacking.

## Summary and future directions

Sequential sampling models are one of the most prominent and comprehensive frameworks for understanding human and monkey decision-making. For nearly four decades, decision behavior has been successfully explained by a standard model that assumes decision makers set a quality criterion before engaging in the decision process and maintain the same criterion throughout. In recent years this assumption of a static criterion has been challenged and a number of authors have suggested that decision makers become increasingly impatient as decision time increases, gradually lowering their quality criterion.

Models with a dynamic decision criterion have been motivated on two grounds. Firstly, decision makers aiming to maximize their reward rate should theoretically adopt a dynamic decision criterion in dynamic environments. Indeed, studies in which the signal-to-noise ratio or the reward for correct decisions varied between or within decisions have shown that models with a dynamic decision criterion can account for the behavior of humans and primates. However, the conclusion that dynamic environments automatically imply a dynamic decision criterion is not uncontested. Many studies purporting such a conclusion did not systematically manipulate the variability of the decision environment. Moreover, quantitative comparisons of how well models with dynamic and static decision criteria can account for data are often missing.

The second main motivation for models with a dynamic decision criterion are single-cell recording studies in behaving monkeys and EEG studies in humans showing patterns of neural activity that are most consistent with a dynamic decision criterion. However, the currently available evidence is equivocal. Neural data from human decision makers are sparse, and theoretical and empirical work linking neural activity at different scales and behavioral outcomes is still missing.

To conclude, the recent developments have led to some enthusiastic responses that have called for models with an impatience component to replace the standard model (Shadlen & Kiani, 2013). Our review of the available evidence indicates that such impatience models certainly provide exciting new impulses for the understanding of decision-making. Nevertheless, the standard model remains a firmly established hallmark of the field and future research efforts will need to delineate more clearly the domain of applicability of each class of models. We now discuss two approaches that will help achieve such a distinction.

### Careful experimentation and quantitative analysis

Future progress in establishing a solid evidence base for models with dynamic decision criteria will critically hinge on careful experimentation in combination with rigorous theoretical analysis. Behavioral and electrophysiological studies will need to systematically manipulate the degree to which a decision environment is dynamic, closely controlling the costs and rewards for decisions and carefully varying the range of signal-to-noise ratios of stimuli. Such environments should be presented to both humans and monkeys, and their behavioral and physiological responses should be compared to models with static and dynamic decision criteria using Bayesian model comparison techniques, which allow researchers not only to determine the best fitting model but also to quantify the uncertainty associated with their conclusions (Jeffreys, 1961; Vandekerckhove, Matzke, & Wagenmakers, 2015). Furthermore, meticulous theoretical analyses will need to quantify the surplus in reward rate obtained by models with dynamic compared to static decision criteria in different environments, thus substantiating often made but rarely tested claims of a general dynamic decision criteria.

A recently developed experimental approach that mitigates the need for computationally intense model fitting (Hawkins, Forstmann, et al., 2015, but see Zhang, Lee, Vandekerckhove, Maris, & Wagenmakers 2014 for a promising new method to fit collapsing thresholds DDMs) are expanded judgment tasks (Vickers, 1979). In these tasks the evidence presented to participants remains available throughout the decision process so that their history of perceptual processing need not be reconstructed computationally but can be easily read out on a moment-to-moment basis. More specifically, the standard experimental paradigm, the random dot motion task, requires participants to extract and accumulate the momentary net motion signal from a noisy stream of information. One consequence of this is that memory leaks might potentially influence the accumulation process, and assumptions about such memory leaks will influence the inferred amount of evidence at decision commitment (Ossmy et al., 2013; Usher & McClelland, 2001), thus complicating comparisons between dynamic and static models. A second consequence is that, as participants are required to extract a motion signal, estimates of the momentary net evidence need to take into consideration the structure of the human visual system (Kiani, Hanks, & Shadlen, 2008; Britten, Shadlen, Newsome, & Movshon, 1993), which even for simplistic approximations amounts to a computationally rather intense problem (Adelson & Bergen, 1985; Watson & Ahumada, 1985). Expanded judgment tasks, on the other hand, allow researchers to reasonably assume that memory leaks play a negligible role because the accumulated evidence is available to participants at all times. Moreover, it is reasonable to assume that participants process information more completely as the rate at which new information is presented is much lower in expanded judgment tasks; indeed, the presented information may be assumed to be analyzed optimally (Brown, Steyvers, & Wagenmakers, 2009). Finally, as expanded judgment tasks usually require numerosity judgments (i.e., decisions as to which part of the visual field contains more items), rather than the extraction of a net motion signal, physiological constraints play a minor role and can easily be approximated by very simple psychophysical laws (Hawkins, Brown, Steyvers, & Wagenmakers, 2012b), so that the participants’ decision criterion can be estimated directly (Brown et al., 2009; Hawkins, Brown, Steyvers, & Wagenmakers, 2012a, b, c). Expanded judgment tasks thus allow the researcher to explicitly test whether the quantity of evidence in the display at the time of response – the decision criterion – decreases as a function of elapsed decision time.

### Linking physiological data on different scales to models

Physiological data will play a pivotal role in discriminating models. Sequential sampling models often make different assumptions about the processes giving rise to decision-making yet predict very similar or even identical behavior (Ditterich, 2010; Purcell et al., 2010; Jones & Dzhafarov, 2014). Physiological recordings allow researchers to directly evaluate such assumptions by comparing the hypothesized evidence accumulation process to neural activity on different scales. On the level of neuron populations, a recently isolated EEG component in humans, the centro-parietal positivity (CPP; O’Connell et al., 2012) holds particularly great promise for physiology-based model comparisons. The CPP seems to be a direct reflection of the evidence accumulation process (Kelly & O’Connell, 2013; O’Connell et al., 2012) and might therefore allow for much more stringent tests of theoretical assumptions than conventional paradigms that attempt to track the accumulated evidence as it is passed on to downstream motor output structures. The CPP might furthermore facilitate comparisons and generalizations across species. In particular, the CPP bears close resemblance to the P3b component (Sutton, Braren, Zubin, & John, 1965), the neural generators of which are most likely located in temporal-parietal areas (Jentzsch & Sommer, 2001; Brázdil, Roman, Daniel, & Rektor, 2003; Polich, 2007), and might thus overlap with areas associated with evidence accumulation in monkeys (Shadlen & Kiani, 2013; Gold & Shadlen, 2007; N. W. D. Thomas & Paré, 2007; Forstmann et al., in press). If EEG-fMRI co-recording studies could indeed link the CPP to the neural generators of the P3b, researchers could obtain recordings with high temporal and spatial resolution of the physiological representation of the accumulated evidence in humans. Comparable recordings in monkeys could then be used not only to establish a correspondence across species, but also to link the evidence accumulation process on the single neuron level to the activity of neuron populations. Such a link could be further corroborated by theoretical work outlining the limitations on the physiological activity patterns at the population level that are consistent with individual accumulators with a dynamic decision criterion.

In sum, the idea of increasing impatience in decision-making has been suggested sporadically throughout the history of sequential sampling models but has seen a tremendous surge in interest over the last years. Although theoretical arguments make a compelling case for impatience, the empirical support from monkey and human data is less clear. Future studies will have to address this problem further and recent developments promise a more conclusive settlement to the debate sooner rather than later. For the time being, we conclude that the idea of impatience has provided novel theoretical impulses, yet reports of the demise of the standard drift diffusion model are greatly exaggerated.

## References

[CR1] Adelson EH, Bergen JR (1985). Spatiotemporal energy models for the perception of motion. Journal of the Optical Society of America. A, Optics and Image Science.

[CR2] Ashby FG (1983). A biased random walk model for two choice reaction times. Journal of Mathematical Psychology.

[CR3] Balci F, Simen P, Niyogi R, Saxe A, Hughes JA, Holmes P, Cohen JD (2011). Acquisition of decision making criteria: Reward rate ultimately beats accuracy. Attention, Perception & Psychophysics.

[CR4] Boehm U, Van Maanen L, Forstmann B, Van Rijn H (2014). Trial-by-trial fluctuations in CNV amplitude reflect anticipatory adjustment of response caution. NeuroImage.

[CR5] Bogacz R, Brown E, Moehlis J, Holmes P, Cohen JD (2006). The physics of optimal decision making: A formal analysis of models of performance in two-alternative forced-choice tasks. Psychological Review.

[CR6] Bowman NE, Kording KP, Gottfried JA (2012). Temporal integration of olfactory perceptual evidence in human orbitofrontal cortex. Neuron.

[CR7] Brázdil M, Roman R, Daniel P, Rektor I (2003). Intracerebral somatosensory event-related potentials: Effect of response type (button pressing versus mental counting) on P3-like potentials within the human brain. Clinical Neurophysiology.

[CR8] Britten KH, Shadlen MN, Newsome WT, Movshon AJ (1992). The analysis of visual motion: A comparison of neuronal and psychophysical performance. Journal of Neuroscience.

[CR9] Britten KH, Shadlen MN, Newsome WT, Movshon AJ (1993). Responses of neurons in macaque MT to stochastic motion signals. Visual Neuroscience.

[CR10] Brown S, Steyvers M, Wagenmakers EJ (2009). Observing evidence accumulation during multi-alternative decisions. Journal of Mathematical Psychology.

[CR11] Busemeyer JR, Rapoport A (1988). Psychological models of deferred decision making. Journal of Mathematical Psychology.

[CR12] Cain N, Shea-Brown E (2012). Computational models of decision making: integration, stability, and noise. Current Opinion in Neurobiology.

[CR13] Cavanagh JF, Wiecki TV, Cohen MX, Figueroa CM, Samanta J, Sherman SJ, Frank MJ (2011). Subthalamic nucleus stimulation reverses mediofrontal influence over decision threshold. Nature Neuroscience.

[CR14] Churchland AK, Kiani R, Chaudhuri R, Wang XJ, Pouget A, Shadlen MN (2011). Variance as a signature of neural computations during decision making. Neuron.

[CR15] Churchland AK, Kiani R, Shadlen MN (2008). Decision-making with multiple alternatives. Nature Neuroscience.

[CR16] Cisek P, Puskas GA, El-Murr S (2009). Decisions in changing conditions: The urgency-gating model. Journal of Neuroscience.

[CR17] Deco G, Rolls ET, Romo R (2009). Stochastic dynamics as a principle of brain function. Progress in Neurobiology.

[CR18] Deneve, S. (2012). Making decisions with unknown sensory reliability. *Frontiers in Neuroscience*, *6*. doi:10.3389/fnins.2012.00075. 10.3389/fnins.2012.00075 PMC3367295 22679418

[CR19] Ditterich J (2006). Evidence for time-variant decision making. The European Journal of Neuroscience.

[CR20] Ditterich J (2006). Stochastic models of decisions about motion direction: Behavior and physiology. Neural Networks.

[CR21] Ditterich, J. (2010). A comparison between mechanisms of multi-alternative perceptual decision making: Ability to explain human behavior, predictions for neurophysiology, and relationship with decision theory. *Frontiers in Neuroscience*, *4*. doi:10.3389/fnins.2010.00184. 10.3389/fnins.2010.00184 PMC2999395 21152262

[CR22] Donner TH, Siegel M, Fries P, Engel AK (2009). Buildup of choice-predictive activity in human motor cortex during perceptual decision making. Current Biology.

[CR23] Drugowitsch J, Moreno-Bote R, Churchland AK, Shadlen MN, Pouget A (2012). The cost of accumulating evidence in perceptual decision making. Journal of Neuroscience.

[CR24] Edwards W (1965). Optimal strategies for seeking information: Models for statistics, choice reaction time, and human information processing. Journal of Mathematical Psychology.

[CR25] Forstmann, B.U., Ratcliff, R., & Wagenmakers, E. (in press). Sequential sampling models in cognitive neuroscience: Advantages, applications, and extensions. *Annual Review of Psychology*. 10.1146/annurev-psych-122414-033645 PMC5112760 26393872

[CR26] Frazier, P.I., & Yu, A.J. (2008). Sequential hypothesis testing under stochastic deadlines. In J. Platt, D. Koller, Y. Singer, & S. Roweis (Eds.), *Advances in Neural Information Processing Systems 20* (pp. 465–472). Cambridge: MIT Press.

[CR27] Gluth S, Rieskamp J, Büchel C (2012). Deciding when to decide: Time-variant sequential sampling models explain the emergence of value-based decisions in the human brain. Journal of Neuroscience.

[CR28] Gluth S, Rieskamp J, Büchel C (2013). Classic EEG motor potentials track the emergence of value-based decisions. NeuroImage.

[CR29] Gluth S, Rieskamp J, Büchel C (2013). Deciding not to decide: Computational and neural evidence for hidden behavior in sequential choice. PLoS Computational Biology.

[CR30] Gold JI, Shadlen MN (2007). The neural basis of decision making. Annual Review of Neuroscience.

[CR31] Gold JI, Shadlen MN, Sales T (2002). Banburismus and the brain: Decoding the relationship between sensory stimuli, decisions, and reward. Neuron.

[CR32] Hanes DP, Schall JD (1996). Neural control of voluntary movement initiation. Science.

[CR33] Hanks TD, Kiani R, Shadlen MN (2014). A neural mechanism of speed-accuracy tradeoff in macaque area LIP. eLife.

[CR34] Hanks TD, Mazurek ME, Kiani R, Hopp E, Shadlen MN (2011). Elapsed decision time affects the weighting of prior probability in a perceptual decision task. Journal of Neuroscience.

[CR35] Hawkins GE, Brown SD, Steyvers M, Wagenmakers E-J (2012). An optimal adjustment procedure to minimize experiment time in decisions with multiple alternatives. Psychonomic Bulletin & Review.

[CR36] Hawkins GE, Brown SD, Steyvers M, Wagenmakers E-J (2012). Context effects in multi-alternative decision making: Empirical data and a Bayesian model. Cognitive Science.

[CR37] Hawkins GE, Brown SD, Steyvers M, Wagenmakers E-J (2012). Decision speed induces context effects in choice. Experimental Psychology.

[CR38] Hawkins GE, Forstmann BU, Wagenmakers E-J, Ratcliff R, Brown SD (2015). Revisiting the evidence for collapsing boundaries and urgency signals in perceptual decision-making. Journal of Neuroscience.

[CR39] Hawkins, G.E., Wagenmakers, E.-J., Ratcliff, R., & Brown, S.D. (2015). Discriminating evidence accumulation from urgency signals in speeded decision making. *Journal of Neurophysiology, 114*(1), 40–47. doi:10.1152/jn.00088.2015. 10.1152/jn.00088.2015 PMC4495756 25904706

[CR40] Heath RA (1981). A tandem random-walk model for psychological discrimination. British Journal of Mathematical and Statistical Psychology.

[CR41] Heath RA (1992). A general nonstationary diffusion model for two-choice decision-making. Mathematical Social Sciences.

[CR42] Heekeren HR, Marrett S, Ungerleider LG (2008). The neural systems that mediate human perceptual decision making. Nature Reviews Neuroscience.

[CR43] Heitz RP, Schall JD (2012). Neural mechanisms of speed-accuracy tradeoff. Neuron.

[CR44] Huang Y, Rao RPN (2013). Reward optimization in the primate brain: A probabilistic model of decision making under uncertainty. PLoS One.

[CR45] Huk AC, Shadlen MN (2005). Neural activity in macaque parietal cortex reflects temporal integration of visual motion signals during perceptual decision making. Journal of Neuroscience.

[CR46] Janssen P, Shadlen MN (2005). A representation of the hazard rate of elapsed time in macaque area LIP. Nature Neuroscience.

[CR47] Jeffreys H (1961). Theory of Probability.

[CR48] Jentzsch I, Sommer W (2001). Sequence-sensitive subcomponents of P300 : Topographical analyses and dipole source localization. Psychophysiology.

[CR49] Jones M, Dzhafarov EN (2014). Unfalsifiability and mutual translatability of major modeling schemes for choice reaction time. Psychological Review.

[CR50] Kelly SP, O’Connell RG (2013). Internal and external influences on the rate of sensory evidence accumulation in the human brain. Journal of Neuroscience.

[CR51] Kiani R, Hanks TD, Shadlen MN (2008). Bounded integration in parietal cortex underlies decisions even when viewing duration is dictated by the environment. The Journal of Neuroscience.

[CR52] Lang W, Cheyne D, Kristeva R, Beisteiner R, Lindinger G, Deecke L (1991). Three-dimensional localization of SMA activity preceding voluntary movement. A study of electric and magnetic fields in a patient with infarction of the right supplementary motor area. Experimental Brain Research.

[CR53] Leon MI, Shadlen MN (2003). Representation of time by neurons in the posterior parietal cortex of the macaque. Neuron.

[CR54] Leuthold H, Jentzsch I (2002). Distinguishing neural sources of movement preparation and execution: An electrophysiological analysis. Biological Psychology.

[CR55] Lo C-C, Boucher L, Paré M, Schall JD, Wang X-J (2009). Proactive inhibitory control and attractor dynamics in countermanding action: A spiking neural circuit model. Journal of Neuroscience.

[CR56] Maddox WT, Bohil CJ (1998). Base-rate and payoff effects in multidimensional perceptual categorization. Journal of Experimental Psychology: Learning, Memory, and Cognition.

[CR57] Mantini D, Hasson U, Betti V, Perrucci MG, Romani GL, Corbetta M, Orban GA, Vanduffel W (2012). Interspecies activity correlations reveal functional correspondence between monkey and human brain areas. Nature Methods.

[CR58] Martin T, Huxlin KR, Kavcic V (2010). Motion-onset visual evoked potentials predict performance during a global direction discrimination task. Neuropsychologia.

[CR59] Miller P, Katz DB (2013). Accuracy and response-time distributions for decision-making: Linear perfect integrators versus nonlinear attractor-based neural circuits. Journal of Computational Neuroscience.

[CR60] Milosavljevic M, Malmaud J, Huth A (2010). The Drift Diffusion Model can account for the accuracy and reaction time of value-based choices under high and low time pressure. Judgment and Decision Making.

[CR61] Mulder MJ, Wagenmakers E-J, Ratcliff R, Boekel W, Forstmann BU (2012). Bias in the brain: A diffusion model analysis of prior probability and potential payoff. Journal of Neuroscience.

[CR62] Niyogi RK, Wong-Lin K (2013). Dynamic excitatory and inhibitory gain modulation can produce flexible, robust and optimal decision-making. PLoS Computational Biology.

[CR63] O’Connell RG, Dockree PM, Kelly SP (2012). A supramodal accumulation-to-bound signal that determines perceptual decisions in humans. Nature Neuroscience.

[CR64] Orban GA, Van Essen D, Vanduffel W (2004). Comparative mapping of higher visual areas in monkeys and humans. Trends in Cognitive Sciences.

[CR65] Ossmy O, Moran R, Pfeffer T, Tsetsos K, Usher M, Donner TH (2013). The timescale of perceptual evidence integration can be adapted to the environment. Current Biology.

[CR66] Petrides M, Tomaiuolo F, Yeterian EH, Pandya DN (2012). The prefrontal cortex: Comparative architectonic organization in the human and the macaque monkey brains. Cortex.

[CR67] Philiastides MG, Sajda P (2006). Temporal characterization of the neural correlates of perceptual decision making in the human brain. Cerebral Cortex.

[CR68] Pike AR (1968). Latency and relative frequency of response in psychophysical discrimination. British Journal of Mathematical and Statistical Psychology.

[CR69] Pitz GF (1968). Information seeking when available information is limited. Journal of Experimental Psychology.

[CR70] Polich J (2007). Updating P300: An integrative theory of P3a and P3b. Clinical Neurophysiology.

[CR71] Purcell BA, Heitz RP, Cohen JY, Schall JD, Logan GD, Palmeri TJ (2010). Neurally constrained modeling of perceptual decision making. Psychological Review.

[CR72] Purcell BA, Schall JD, Logan GD, Palmeri TJ (2012). From salience to saccades: Multiple-alternative gated stochastic accumulator model of visual search. Journal of Neuroscience.

[CR73] Rao, R.P.N. (2010). Decision making under uncertainty: A neural model based on partially observable markov decision processes. *Frontiers in Computational Neuroscience*, *4*. doi:10.3389/fncom.2010.00146. 10.3389/fncom.2010.00146 PMC2998859 21152255

[CR74] Rapoport A, Burkheimer GJ (1971). Models for deferred decision making. Journal of Mathematical Psychology.

[CR75] Ratcliff R (1978). A theory of memory retrieval. Psychological Review.

[CR76] Ratcliff, R., Hasegawa, Y.T., Hasegawa, R.P., Childers, R., Smith, P.L., & Segraves, M.A. (2011). Inhibition in superior colliculus neurons in a brightness discrimination task? *Neural Computation, 23*, 1790–1820. 10.1162/NECO_a_00135 PMC4963236 21492006

[CR77] Ratcliff, R., Hasegawa, Y.T., Hasegawa, R.P., Smith, P.L., & Segraves, M.A. (2007). Dual diffusion model for single-cell recording data from the superior colliculus in a brightness-discrimination task. *Journal of Neurophysiology, 97*, 1756–1774. 10.1152/jn.00393.2006 PMC2394732 17122324

[CR78] Ratcliff R, McKoon G (2008). The diffusion decision model: Theory and data for two-choice decision tasks. Neural Computation.

[CR79] Ratcliff R, Philiastides MG, Sajda P (2009). Quality of evidence for perceptual decision making is indexed by trial-to-trial variability of the EEG. Proceedings of the National Academy of Sciences.

[CR80] Ratcliff R, Smith PL (2004). A comparison of sequential sampling models for two-choice reaction time. Psychological Review.

[CR81] Resulaj A, Kiani R, Wolpert DM, Shadlen MN (2009). Changes of mind in decision-making. Nature.

[CR82] Roitman JD, Shadlen MN (2002). Response of neurons in the lateral intraparietal area during a combined visual discrimination reaction time task. Journal of Neuroscience.

[CR83] Sanders AF, Ter Linden W (1967). Decision making during paced arrival of probabilistic information. Acta Psychologica.

[CR84] Shadlen MN, Kiani R (2013). Decision making as a window on cognition. Neuron.

[CR85] Shadlen MN, Newsome WT (2001). Neural basis of a perceptual decision in the parietal cortex (area LIP) of the rhesus monkey. Journal of Neurophysiology.

[CR86] Siegel, M., Engel, A.K., & Donner, T.H. (2011). Cortical network dynamics of perceptual decision-making in the human brain. *Frontiers in Human Neuroscience*, *5*. doi:10.3389/fnhum.2011.00021. 10.3389/fnhum.2011.00021 PMC3047300 21427777

[CR87] Simen P, Contreras D, Buck C, Hu P, Holmes P, Cohen JD (2009). Reward rate optimization in two-alternative decision making: Empirical tests of theoretical predictions. Journal of Experimental Psychology: Human Perception and Performance.

[CR88] Smith PL (1995). Psychophysically principled models of visual simple reaction time. Psychological Review.

[CR89] Smith PL (2010). From Poisson shot noise to the integrated Ornstein-Uhlenbeck process: Neurally principled models of information accumulation in decision-making and response time. Journal of Mathematical Psychology.

[CR90] Standage, D., You, H., Wang, D.-H., & Dorris, M.C. (2011). Gain modulation by an urgency signal controls the speed-accuracy trade-off in a network model of a cortical decision circuit. *Frontiers in Computational Neuroscience*, *5*. doi:10.3389/fncom.2011.00007. 10.3389/fncom.2011.00007 PMC3042674 21415911

[CR91] Starns JJ, Ratcliff R (2010). The effects of aging on the speed-accuracy compromise: Boundary optimality in the diffusion model. Psychology and Aging.

[CR92] Starns JJ, Ratcliff R (2012). Age-related differences in diffusion model boundary optimality with both trial-limited and time-limited tasks. Psychonomic Bulletin & Review.

[CR93] Stone M (1960). Models for choice-reaction time. Psychometrika.

[CR94] Summerfield, C., & Tsetsos, K. (2012). Building bridges between perceptual and economic decision-making: Neural and computational mechanisms. *Frontiers in Neuroscience*, *6*. doi:10.3389/fnins.2012.00070. 10.3389/fnins.2012.00070 PMC3359443 22654730

[CR95] Sutton S, Braren M, Zubin J, John E (1965). Evoked potential correlates of stimulus uncertainty. Science.

[CR96] Swensson RG, Thomas RE (1974). Fixed and optional stopping models for two-choice discrimination times. Journal of Mathematical Psychology.

[CR97] Thomas NWD, Paré M (2007). Temporal processing of saccade targets in parietal cortex area LIP during visual search. Journal of Neurophysiology.

[CR98] Thura D, Beauregard-Racine J, Fradet C-W, Cisek P (2012). Decision making by urgency gating: Theory and experimental support. Journal of Neurophysiology.

[CR99] Thura D, Cisek P (2014). Deliberation and commitment in the premotor and primary motor cortex during dynamic decision making. Neuron.

[CR100] Thura D, Cos I, Trung J, Cisek P (2014). Context-dependent urgency influences speed-accuracy trade-offs in decision-making and movement execution. Journal of Neuroscience.

[CR101] Usher M, McClelland JL (2001). The time course of perceptual choice: The leaky, competing accumulator model. Psychological Review.

[CR102] Van Vugt, M.K., Simen, P., Nystrom, L.E., Holmes, P., & Cohen, J.D. (2012). EEG oscillations reveal neural correlates of evidence accumulation. *Frontiers in Neuroscience*, *6*. doi:10.3389/fnins.2012.00106. 10.3389/fnins.2012.00106 PMC3398314 22822389

[CR103] Vandekerckhove J, Matzke D, Wagenmakers E-J, Busemeyer J, Townsend J, Wang ZJ, Eidels A (2015). Model comparison and the principle of parsimony. Oxford Handbook of Computational and Mathematical Psychology.

[CR104] Vickers, D. (1979). *Decision Processes in Visual Perception*. London: Academic Press.

[CR105] Wallsten TS (1968). Failure of predictions from subjectively expected utility theory in a Bayesian decision task. Organizational Behavior and Human Performance.

[CR106] Wang X-J (2002). Probabilistic decision making by slow reverrberation in cortical circuits. Neuron.

[CR107] Watson AB, Ahumada AJ (1985). Model of human visual-motion sensing. Journal of the Optical Society of America. A, Optics and Image Science.

[CR108] Winkel J, Keuken MC, Van Maanen L, Wagenmakers E-J, Forstmann BU (2014). Early evidence affects later decisions: Why evidence accumulation is required to explain response time data. Psychonomic Bulletin & Review.

[CR109] Wyart V, de Gardelle V, Scholl J, Summerfield C (2012). Rhythmic fluctuations in evidence accumulation during decision making in the human brain. Neuron.

[CR110] Zandbelt B, Purcell BA, Palmeri TJ, Logan GD, Schall JD (2014). Response times from ensembles of accumulators. Proceedings of the National Academy of Sciences.

[CR111] Zhang, S., Lee, M.D., Vandekerckhove, J., Maris, G., & Wagenmakers, E.-J. (2014). Time-varying boundaries for diffusion models of decision making and response time. *Frontiers in Psychology*, *5*. doi:10.3389/fpsyg.2014.01364. 10.3389/fpsyg.2014.01364 PMC4260487 25538642

